# Influence of Interval Training Frequency on Time-Trial Performance in Elite Endurance Athletes

**DOI:** 10.3390/ijerph17093190

**Published:** 2020-05-04

**Authors:** Espen Tønnessen, Jonny Hisdal, Bent R. Ronnestad

**Affiliations:** 1 School of Health Sciences, Kristiana University College, 0153 Oslo, Norway; 2 Department of Vascular Investigations, Oslo University Hospital—Aker, 0586 Oslo, Norway; jonny.hisdal@medisin.uio.no; 3 Section for Health and Exercise Physiology, Inland Norway University, 2624 Lillehammer, Norway; bent.ronnestad@inn.no

**Keywords:** cross-country skiers, exercise economy, VO_2max_

## Abstract

Purpose: To determine the impact of interval training frequency in elite endurance athletes. It was hypothesized that two longer sessions would elicit greater performance improvements and physiological adaptation than four shorter sessions at the same intensity. Methods: Elite cross-country skiers and biathletes were randomly assigned to either a high-frequency group (HF group) (5 M, 1 F, age 22 (19–26), VO_2max_ 67.8 (65.5–70.2) mL/kg/min) doing four short interval sessions per week or a low-frequency group (LF group) (8 M, 1 F, age 22 (18–23), VO_2max_ 70.7 (67.0–73.9) mL/kg/min) doing two longer interval sessions. All interval sessions were performed at ~85% of maximum heart rate, and groups were matched for total weekly training volume. Pre- and post-intervention, athletes completed an 8 km rollerski time-trial, maximal oxygen uptake (VO_2max_) test, and an incremental, submaximal exercise test. Results: The LF group had a statistically significant improved time-trial performance following the intervention (*p* = 0.04), with no statistically significant changes in the HF group. Similarly, percentage utilization of VO_2max_ at anaerobic threshold (*p* = 0.04) and exercise economy (*p* = 0.01) were statistically significantly improved following the intervention in the LF group only. No statistically significant changes in VO_2max_ were observed in either group. Conclusions: Two longer interval sessions appear superior to four shorter sessions per week in promoting endurance adaptations and performance improvements in elite endurance athletes. Despite matched training volume and exercise intensity, the larger, more concentrated exercise stimulus in the LF group appears to induce more favorable adaptations. The longer time between training sessions in the LF group may also have allowed athletes to recover more effectively and better “absorb” the training. These findings are in line with the “best practice” observed by many of the world’s best endurance athletes.

## 1. Introduction

At a senior level, endurance athletes frequently train up to 1000 h per year, of which 80%–90% is typically conducted at low intensity [1,2,3]. The remaining 10%–20% is composed of high-intensity training in the form of competition, high-intensity continuous training, and interval training.

The principle of interval training was first described by Reindell & Roskamm in 1959 [4] and is a method of training which alternates between exercise periods with high and low intensity. Compared to continuous exercise, interval training allows the athlete to maintain a higher intensity and work for longer time at high intensities (>85% maximal heart rate (HRmax)) [5,6,7,8]. Increased training time at high intensity creates better conditions to optimally stimulate the development of performance capacity, maximal oxygen uptake (VO_2max_), percentage utilization of maximal oxygen uptake (% VO_2max_) and exercise economy [9,10].

Six factors determine total workload: duration per interval, exercise intensity, recovery duration, recovery intensity, movement patterns, and total training time [11,12]. Depending on the purpose of the training session, these variables can be manipulated to give countless different combinations. Studies of well-trained endurance athletes indicate that the best training effect, i.e., increased stroke volume and oxygen delivery, is achieved at intensities between 85%–95% of HRmax [13,14,15], although studies have also demonstrated positive effects of training at higher intensities [12,16].

The optimal interval duration for well-trained endurance athletes appears to be 4–10 min [13,14,15], with a beneficial effect observed at a total effective duration of approximately 15 min [5,13]. However, durations of 30–45 min appear to elicit the best effect [14,15].

The optimal number of high-intensity interval training sessions per week in order to maximally stimulate the development of performance has not yet been elucidated. In a retrospective study it was found that world-class endurance athletes performed 2–3 high-intensity training sessions per week (100–140 sessions per year) [17]. Half of these sessions were completed as interval training, with 30%–50% of these performed at an intensity around 85% HR_max,_ with a total effective duration of 30–75 min per session [17].

The aim of the present study was therefore to determine the effect of frequency of high-intensity interval training in elite cross-country skiers. Based on the “best practice” observed in world-class endurance athletes, our hypothesis was that two longer interval sessions at an intensity of ~85% HR_max_ would elicit greater improvements in time-trial performance, VO_2max_, %VO_2max_ at anaerobic threshold (AT), and work economy than four shorter sessions at the same intensity.

## 2. Materials and Methods

### 2.1. Subjects

Twenty elite cross-country skiers and biathletes volunteered their written, informed consent to take part in the study, which was approved by the institution’s ethics committee, in accordance with the Declaration of Helsinki; the study was further approved by the Norwegian center for research data (number: 26732). All participants had competed in cross-country skiing or a biathlon at a national level for a minimum of three years. Athletes were randomly assigned to either a high-frequency group (HF group, *n* = 10) doing four interval sessions per week or a low-frequency group (LF group, *n* = 10) doing two longer interval sessions. There was one drop-out in the LF group due to illness. There were four drop-outs in the HF group, three due to illness and one to an accident not related to the project. The athletes’ physiological and anthropometric characteristics and initial training status are shown in Table 1.

### 2.2. Procedures

**Figure 1:**
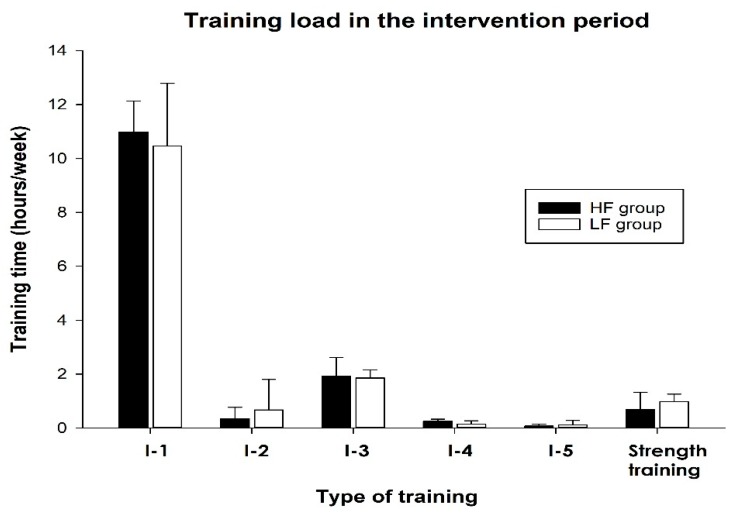
Weekly training load in the 12-week intervention period. The figure shows the distribution of endurance training in the different intensity zones (I-1 to I-5) and duration of strength training in the high- and low-frequency group (HF- and LF group). Values are presented as the median and IQR. There were no statistically significant differences between the groups.

During the 12-week intervention period, the LF group performed two interval sessions per week, whilst the HF group performed four sessions per week, at the same intensity and matched for weekly training volume (Figure 1). Oxygen consumption (VO_2_), heart rate (HR), and blood lactate were measured, and exercise economy was calculated pre- and post-intervention. In addition, a performance test (8 km roller-skiing time trial) was conducted before and after the intervention period.

#### 2.2.1. Interval Training

The intervention was performed as dry land training during the summer period from May to August. The exercise intensity scale developed by the Norwegian Olympic Centre (NOC) was used for classification of training intensity (Table 2). Interval durations and work intensity for both interventions were selected to correspond with what the literature suggests to be an effective model to elicit performance adaptations in endurance athletes.

Outside of the planned intervention sessions, athletes were instructed to train normally, with low-intensity training (<82% HR_max_ I-zones 1–2) and a maximum of one additional session per week of high-intensity interval training (>87% HR_max_, I-zones 4–5). To ensure that the training load during the intervention period outside of prescribed training did not differ between groups, subjects were provided with an electronic training diary with both written and verbal instructions on how to record their training. Data recorded in these diaries were used to calculate the training volume and intensity distribution.

During the intervention period, the LF group completed one session per week of 8 × 8 min intervals in I-zone 3 with 2 min recoveries (total time 64 min) and one session of 6 × 12 min intervals in I-zone 3 with 3 min recoveries (total time 72 min). The HF group performed twice-weekly sessions of 4 × 8 min in I-zone 3 separated by 2 min recoveries (total time 32 min) and two sessions of 3 × 12 min in I-zone 3 with 3 min recoveries (total time 36 min). All interval sessions were performed as rollerski skating. For both groups, the total prescribed weekly training time in I-zone 3 was 136 min. The LF group had a minimum of one day, and typically two days, between each session in I-zone 3. The HF group performed two I-zone 3 sessions on two consecutive days, while there was one day off before the remaining I-zone 3 session.

Each interval session included a self-selected warm-up and warm-down. During the first four weeks of the intervention period, 1–2 interval sessions per week were organized as group training sessions, during which blood lactate and HR were monitored and controlled to ensure that athletes were training at the planned intensity. During the remaining weeks of the intervention, once participants had become accustomed to maintaining the correct intensity during training sessions, only a selection of sessions were monitored.

#### 2.2.2. Test Day 1: Submaximal Exercise Test and VO_2max_

Pre- and post-intervention, subjects performed a submaximal incremental rollerskiing (skating) test on a motorized treadmill (Lode Valiant Special, Lode B.V. Groningen, Netherlands) in a laboratory maintained at 17–21 °C and 25%–40% relative humidity. Participants completed a ~15-min standardized warm-up. The test started at 5% incline and 10.8 km/h for men and 9 km/h for women. Each stage lasted 5 min, followed by a one-minute recovery. The treadmill incline was increased by 2% between each stage. The test was terminated when blood lactate concentration exceeded 4.0 mmol/L (Lactate Pro LT-1710t, ArkRay Inc., Kyoto, Japan). Expired air was analyzed continuously with VO_2_ determined at 30 s intervals throughout the test (Oxycon Pro, Erich Jaeger, Hoechberg, Germany). HR was measured continuously using short-range telemetry (Polar S620i, Kempele, Finland). For both HR and VO_2_, the average values recorded during the final three minutes of each stage were used for analyses. A finger-prick blood sample was taken during the recovery between each stage for blood lactate determination. Exercise economy was calculated from body mass-adjusted oxygen consumption, and HR and lactate recorded for the lowest test workload (5% incline 10.8 km/h for men and 9 km/h for women). This workload was chosen to ensure aerobic metabolism, indicated by a lactate level < 2.5 mmol/L.

VO_2_ at an estimated lactate concentration of 4.0 mmol/L was calculated by a forecast algorithm. We used a built-in algorithm in Excel 2016, using the measured values beyond and above an intensity corresponding to 4.0 mmol/L to predict a given *y*-value (VO_2_) at a given *x*-value (4.0 mmol/L), based on the measured values in the data set, using the algorithm *a* + *bx*:

(1) $$a=\bar{y}-b\bar{x}\;\mathrm{and}\;b=\frac{\sum \left(x-\bar{x}\right)\left(y-\bar{y}\right)}{\sum {\left(x-\bar{x}\right)}^{2}}$$

Here, *x* and *y* are the sample means average (*x* = lactate, *y* = VO_2_).

Following ~10 min recovery, VO_2max_ was measured via a continuous, incremental rollerskiing (skating) test to volitional exhaustion. The test started at a 5% incline and 10.8 km/h for men and 9 km/h for women. Every minute, the treadmill incline was increased by 2%. When an incline of 15% was reached, treadmill speed was then increased by 0.5 km/h every minute. VO_2_ was measured continuously, and the average of the two highest consecutive 30 s measurements was defined as VO_2max_. Respiratory exchange ratio ≥ 1.05, a plateau in VO_2_ with increasing workload, and blood lactate concentration ≥ 8 mmol/L were used as criteria to evaluate if VO_2max_ was obtained [18]. HR was measured continuously, with the highest value during the test defined as HRpeak.

#### 2.2.3. Test Day 2: Rollerski Time Trial Performance

Pre- and post-intervention, each athlete completed an 8 km rollerski (skating) uphill time-trial outdoors. This had a typical duration of 30–35 min and was preceded by a 30 min standardized warm-up. Athletes started in a random order at 30 s intervals pre-intervention, and again in this same order post-intervention to prevent any possible motivational effects. Performance times were recorded by two synchronized stopwatches (Regnly RT3, Emit AS, Oslo, Norway). The weather conditions pre- and post-intervention were stable, with ambient temperature between 17–20 °C, no wind and dry asphalt. All athletes were familiar with the 8 km course.

Subjects used identical rollerskis (Swenor Skate, Sport Import AS. Sarpsborg, Norway), with each athlete using the same pair of skis pre- and post-intervention. These were not used between pre- and post-testing to avoid changes in rolling resistance. Participants also used the same skating poles (SWIX CT3, Swix Sport AS. Lillehammer, Norway) of self-selected length. The two test days were separated by a minimum of 48 h and a maximum of 6 days. Prior to testing, participants underwent a standardized 2 day tapering period. Tests were performed at the same time of day (± 1 h) and following a 1 h fast. Participants were required to abstain from strenuous exercise, alcohol, and caffeine during the 24 h leading up to each test. Athletes not able to participate in whole intervention period were excluded from the study (*n* = 1 in the LF group and *n* = 4 in the HF group).

### 2.3. Statistical Analyses

Since not all variables passed the normality test, all results are presented as median values with the corresponding inter-quartile range (IQR). A Wilcoxon matched-pairs signed rank test was used to test for differences between each parameter before and after the 12-week intervention period. In all comparisons, two-tailed tests were used. Differences were considered significant at *p* < 0.05. Due to the small sample size and expectations of small changes in these already well-trained athletes, the data were further analyzed with mean effects size (ES). ES was calculated as Cohen’s d to compare the practical significance of the performance improvements among the two groups. The criteria to interpret the magnitude of the ES were 0.0–0.2 trivial, 0.2–0.6 small, 0.6–1.2 moderate, 1.2–2.0 large, and >2.0 very large. The calculations were performed in Microsoft Excel 2010 (Microsoft Corporation), and the statistical analyses were conducted in SigmaPlot for Windows, version 12.2 (Systat Software GmbH, Erkrath, Germany).

## 3. Results

All included test subjects completed > 80% of the interval sessions and no statistically significant differences were observed in training compliance between the groups. When prescribed interval sessions were skipped, that was mainly due to the athletes requesting a rest due to a feeling of tiredness.

### 3.1. Baseline

At baseline, no statistically significant differences were observed between the LF- and HF group with respect to age, height, weight, VO_2max_ or training volume (Table 1). Further, there were no statistically significant differences in exercise economy, %VO_2max_, %VO_2max_ at 4 mmol/L lactate or time-trial performance.

### 3.2. Training Load in the Intervention Period

There was no difference in the weekly training load between groups in terms of training volume, intensity distribution, or resistance training during the intervention period (Figure 1). Despite a higher median training load in both the last 12 and last 2 months, the statistical analysis revealed that there was no systematic differences between the groups. In our opinion, it is therefore unlikely that difference in training volume had a significant impact on the results.

### 3.3. Time-Trial Performance

**Figure 2:**
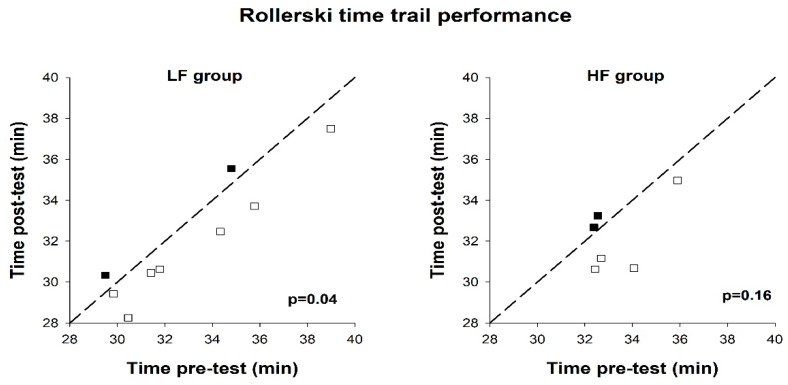
Time taken to complete the 8 km rollerski time-trial, pre-test and post-test, in the low-frequency group (LF group) and high-frequency group (HF group). Reference lines indicate identical values pre- and post-test. Each box represents a test subject. Open boxes indicate an improvement, and filled boxes indicate a decline, compared to the pre-test.

In the LF group, seven out of nine subjects showed an improvement in rollerski time-trial performance following the intervention, with median time-trial time that was statistically significantly lower at post-intervention (30.6 (30.1–34.1) min vs. 31.8 (30.3–35.0) min, *p* = 0.04). In the HF group, no statistically significant improvement between pre- and post-test was observed (*p* = 0.16). ES of the changes revealed only a trivial effect of LF training vs. HF training (ES = 0.17). Individual results from the time-trial performance test are shown in Figure 2.

### 3.4. VO_2max_ and % VO_2max_ at AT

**Figure 3:**
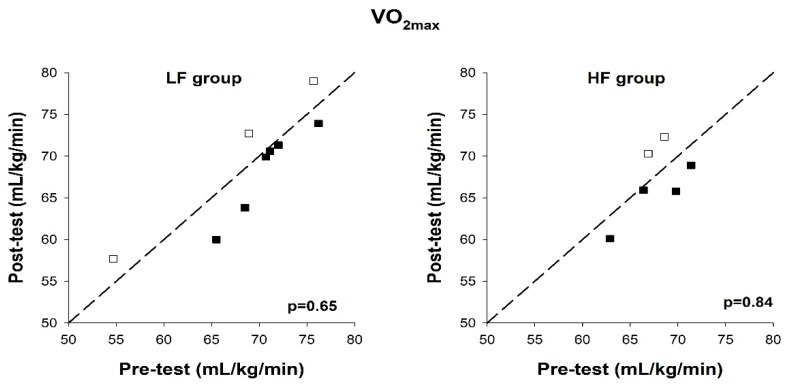
VO_2max_ at pre-test and post-test, in the low-frequency group (LF group) and high-frequency group (HF group). Reference lines indicate identical values pre- and post-test. Each box represents a test subject. Open boxes indicate an improvement and filled boxes indicate a decline, compared to the pre-test.

**Figure 4:**
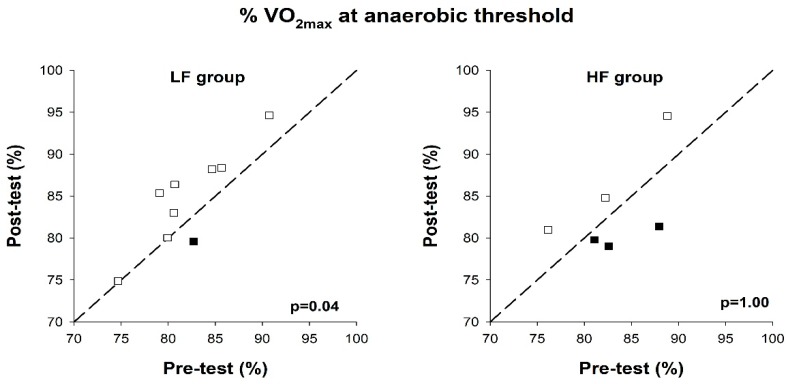
% of VO_2max_ at anaerobic threshold at pre-test and post-test, in the low-frequency group (LF group) and high-frequency group (HF group). Reference lines indicate identical values at pre- and post-test. Each box represents a test subject. Open boxes indicate an improvement and filled boxes indicate a decline, compared to the pre-test.

No statistically significant changes in VO_2max_ were observed between pre- and post-intervention in either group. Individual values for all subjects are shown in Figure 3. There was no statistically significant change in body mass in either group. % VO_2max_ at anaerobic threshold (lactate 4.0 mmol/L) was statistically significantly increased after the intervention period in the LF group (85.4 (79.9–88.2)% vs. 80.7 (79.7–84.9)%, *p* = 0.04) but not in the HF group (*p* = 1.00). Individual results are shown in Figure 4. The mean ES revealed a small practical effect of the LF training vs. HF training (ES = 0.52).

### 3.5. Exercise Economy

**Figure 5:**
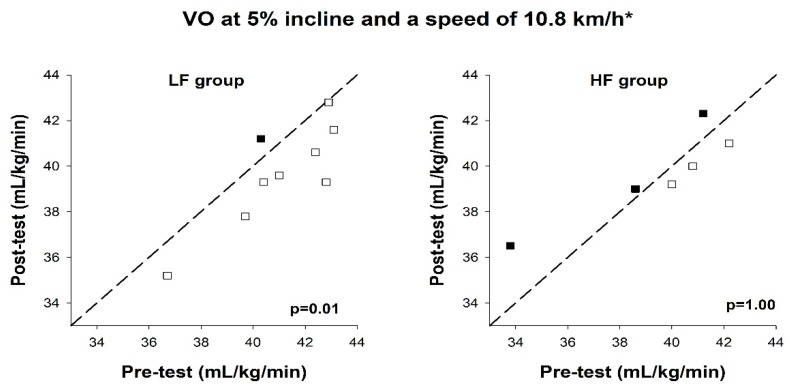
VO_2_ at 5% incline and a speed of 10.8 km/h at pre-test and post-test, in the low-frequency group (LF group) and high-frequency group (HF group). Reference lines indicate identical values at pre- and post-test. Each box represents a test subject. Open boxes indicate an improvement and filled boxes indicate a decline, compared to the pre-test. * = 9.0 km/h for women.

A statistically significant reduction in submaximal oxygen consumption at 10.9 km/h and 5% incline was observed in the LF group (39.6 (39.3–41.2) mL/kg/min vs. 41.0 (40.3–42.8) mL/kg/min, *p* = 0.01), but not in the HF group at post-test. Individual values are shown in Figure 5. The ES showed a moderate practical effect of the LF training vs. HF training on improvements in exercise economy (ES = 1.14).

**Figure 6:**
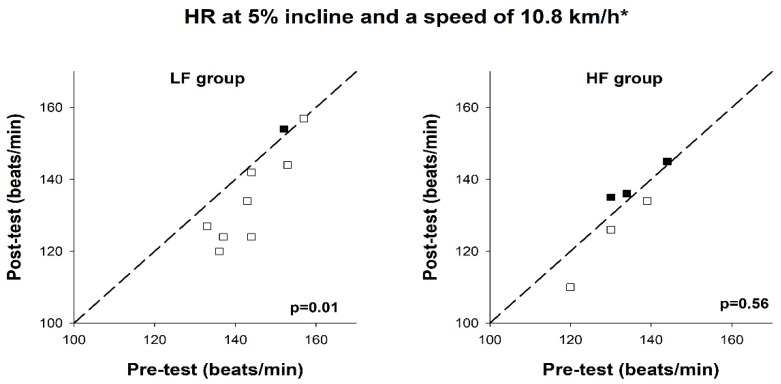
Heart rate at a speed of 10.8 km/h at pre- and post-test, in the low-frequency group (LF group) and high-frequency group (HF group). Reference lines indicate identical values at pre- and post-test. Each box represents a test subject. Open boxes indicate an improvement and filled boxes indicate a decline, compared to the pre-test. * = 9.0 km/h for women.

There was a statistically significant reduction in submaximal HR for the LF group (134 (124–144) vs. 144 (137–152) bpm, *p* = 0.01) but not for the HF group. Individual results are demonstrated in Figure 6. Mean ES of the improvement in submaximal HR revealed a moderate practical effect of performing LF training vs. HF training (ES = 0.95).

At the lowest workload, blood lactate for all subjects was <2.5 mmol/L both pre- and post-intervention. There were no statistically significant differences observed for either group.

## 4. Discussion

The main finding in the present study is that highly trained cross-country skiers and biathletes performing two longer high-intensity interval sessions per week (LF group) for a period of 12 weeks, showed a statistically significant improvement in 8 km rollerski time-trial performance. These improvements can likely be explained by a concurrent improvement in % VO_2max_ at AT and exercise economy. There were no statistically significant improvements in rollerski time-trial performance, % VO_2max_ at AT or exercise economy in the HF group, who performed the same weekly volume of high-intensity training as the LF group but distributed among four shorter sessions.

### 4.1. Rollerski Time-Trial Performance

The improvement in the time-trial performance in the LF group is in line with that presented by Seiler et al. [15], showing that training around the anaerobic threshold is effective in improving long- duration endurance exercise performance. It is possible to speculate that significant improvements in performance may not have been seen if a shorter time-trial had been used, whereas improvements may have been even more pronounced with a longer duration test.

The external conditions such as asphalt, weather conditions, and competitive element of the task and roller ski equipment were similar pre-and post-intervention and should therefore not have impacted the time-trial results.

### 4.2. VO_2max_ Test

The present findings of no statistically significant change in VO_2max_ in either group is in accordance with findings from previous studies with well-trained cross-country skiers following a similar length training intervention [19,20]. The most likely explanation for this is probably that all the athletes had a long training history, with many years of training at a high level. With prolonged training, improvements in VO_2max_ eventually plateau and there is some evidence for a genetic “ceiling”, making it difficult to further increase VO_2max_ in already highly-trained endurance athletes [21,22]. Another possible explanation is that the interval training sessions used in the current study were not of a sufficiently high exercise intensity to stimulate improvements in VO_2max_. Previous studies have shown that training at an intensity above the anaerobic threshold is necessary in order to stimulate an increase in VO_2max_ in trained athletes [13,15].

### 4.3. Submaximal Rollerskiing

The increase in %VO_2max_ at AT in the LF group is in line with our hypothesis. However, other studies finding improved aerobic endurance performance reported no changes or minor improvements in %VO_2max_ at AT in endurance trained athletes [13,23]. A possible explanation for this may be differences in the length of the intervention period, interval duration or effective training time in I-zone 3.

Despite equivalent total training volume and intensity, the longer duration per session in the LF group may better stimulate peripheral adaptations such as capillarization, increased size and number of mitochondria, and increased activity of enzymes involved in aerobic respiration [24]. The main stimulus for exercise-induced capillarization in skeletal muscle is increased shear stress due to reactive hyperemia and high strain (e.g., muscle stretch; reviewed by Egginton [25]). It has therefore been suggested that in well-trained endurance athletes, a larger and more concentrated exercise stimulus leads to a higher level of capillarization [26]. As the effective duration in I-zone 3 in the LF group was 64–72 min per session compared to 32–36 min in the HF group, athletes in the LF group were exposed to a larger and more concentrated exercise stimulus. Hence, improved capillarization may partly explain the finding of improved exercise economy in the LF group only. It has been suggested that gross efficiency is improved with high intensity training [27]. Furthermore, it has recently been observed that a higher rate of glycogen utilization during exercise is associated with increased activation of intracellular signaling cascades central in mitochondrial biogenesis [28]. Despite these speculations, the latter may suggest a potential superiority of an exercise session with large glycogen utilization, and theoretically, there was a larger glycogen consumption in the LF training compared to the HF training [29]. It has previously been reported that training in a state of low muscle glycogen to a greater degree stimulates adaptation to endurance exercise [30].

Improved exercise economy in the LF group likely explains the concurrent reduction in submaximal HR. The observed improvement in exercise economy should, in theory, have been coupled with reduced submaximal blood lactate concentration in the LF group. However, this was not the case at the specific workload at which exercise economy was measured in the current study. This is likely related to the aerobic nature of this workload and low blood lactate values at pre-intervention making it difficult to detect changes.

At inclusion, group sizes were matched. However, due to greater drop-out in the HF group, the statistical power of the study was slightly reduced. The present study is too small and it was not decided to elucidate potential differences in adherence to LF- vs. HF-interventions. Recruiting a sufficient number of participants becomes challenging when conducting research with elite athletes. However, we considered as a strength of the study that highly trained athletes were included, rather than sedentary or recreationally active participants. It should be noted that even though there were no differences between groups in weekly training duration in I-zone 3, the difference in the number of work intervals during each session probably induced differences between groups in the session rate of perceived exertion. Unfortunately, the rate of perceived exertion during the sessions was not recorded in the present study.

### 4.4. Practical Applications and Limitation of the Study

The results of the current study indicate that in order to stimulate improvements in rollerski time-trial performance, %VO_2max_ at AT, and exercise economy in elite endurance athletes, an interval training model with lower training frequency but longer duration per session appears to be more effective. Interval sessions in I-zone 3 appear to elicit the best effect when the total effective duration is ≥60 min. Regardless of whether the total training load remains the same, more frequent sessions of a shorter duration appear less effective in stimulating peripheral adaptations and performance improvements. These findings may be particularly applicable to endurance athletes competing in sports with a long competition time, as previous literature indicates that training at this intensity is particularly beneficial to prolonged exercise performance. One of the limitations of the present study was the number of participants (*n* = 20). However, it is very difficult to achieve high number of participants from individual sports and therefore we believe that this study could contribute to future meta-analysis studies in the field.

## 5. Conclusions

In a group of elite endurance athletes, two long interval sessions appeared to be more effective than four shorter sessions per week in promoting endurance adaptations and performance improvements. Despite both groups performing the same training volume at the same exercise intensity, it appears that the larger and more concentrated exercise stimulus in the LF group induced more favorable endurance adaptations. The longer time between training sessions in the LF group may have allowed the athletes to recover better and more effectively “absorb” the training. These findings are in line with the current endurance training philosophy in Norwegian elite sport and with the “best practice” observed by many of the world’s best endurance athletes [2,3].

## Figures and Tables

**Table 1:** Baseline characteristics of test subjects in the high-frequency group (HF group) performing four interval sessions per week, and in the low-frequency group (LF group) that performed two interval sessions per week. Values are presented as the median and inter quartile range (IQR). There were no significant differences between the groups.

Characteristics of the Subjects	HF Group	LF Group
Gender	♂ = 5, ♀ = 1	♂ = 8, ♀ = 1
Age (years)	22.0 (18.8–26.0)	22.0 (18.0–22.5)
Height (m)	1.81 (1.75–1.86)	1.82 (1.80–1.84)
Body mass (kg)	72.3 (64.6–78.0)	76.9 (72.1–79.9)
VO_2max_ (mL/min/kg)	67.8 (65.5–70.2)	70.7 (67.0–73.9)
Training volume last 12 months (hours)	565 (431–638)	650 (520–755)
Training volume last 2 months (hours)	65 (58–81)	75 (63–80)

**Table 2:** The exercise intensity scale developed by the Norwegian Olympic Centre (NOC) used for classification of training intensity (I-zones).

Intensity Zone	% of HR_max_	Blood Lactate (mmol/L) *	Examples of Training Models
I-zone 5	92–100	6.0–10.0	Interval training with maximal or near maximal exertion. Recovery period equivalent to 70%–90% of the work interval time.
I-zone 4	87–92	4.0–6.0	High-intensity continuous training or intervals with a high level of exertion. Recovery periods equivalent to approximately 50% of the work time.
I-zone 3	82–87	2.5–4.0	Natural interval training, intensive continuous training, or long intervals. Recovery periods equivalent to 20%–30% of the work time.
I-zone 2	72–82	1.5–2.5	Moderate intensity continuous work.
I-zone 1	55–72	<1.5	Recovery sessions and low-intensity continuous work.

* Values determined via a hand-held or portable lactate analyzer using red cell lysed blood.
